# JTT-130, a microsomal triglyceride transfer protein (MTP) inhibitor lowers plasma triglycerides and LDL cholesterol concentrations without increasing hepatic triglycerides in guinea pigs

**DOI:** 10.1186/1471-2261-5-30

**Published:** 2005-09-27

**Authors:** Dimple Aggarwal, Kristy L West, Tosca L Zern, Sudeep Shrestha, Marcela Vergara-Jimenez, Maria Luz Fernandez

**Affiliations:** 1 Department of Nutritional Sciences, University of Connecticut, Storrs, CT, USA; 2 Department of Nutritional Sciences, University of Sinaloa, Culiacan, Mexico

## Abstract

**Background:**

Microsomal transfer protein inhibitors (MTPi) have the potential to be used as a drug to lower plasma lipids, mainly plasma triglycerides (TG). However, studies with animal models have indicated that MTPi treatment results in the accumulation of hepatic TG. The purpose of this study was to evaluate whether JTT-130, a unique MTPi, targeted to the intestine, would effectively reduce plasma lipids without inducing a fatty liver.

**Methods:**

Male guinea pigs (n = 10 per group) were used for this experiment. Initially all guinea pigs were fed a hypercholesterolemic diet containing 0.08 g/100 g dietary cholesterol for 3 wk. After this period, animals were randomly assigned to diets containing 0 (control), 0.0005 or 0.0015 g/100 g of MTPi for 4 wk. A diet containing 0.05 g/100 g of atorvastatin, an HMG-CoA reductase inhibitor was used as the positive control. At the end of the 7^th ^week, guinea pigs were sacrificed to assess drug effects on plasma and hepatic lipids, composition of LDL and VLDL, hepatic cholesterol and lipoprotein metabolism.

**Results:**

Plasma LDL cholesterol and TG were 25 and 30% lower in guinea pigs treated with MTPi compared to controls (P < 0.05). Atorvastatin had the most pronounced hypolipidemic effects with a 35% reduction in LDL cholesterol and 40% reduction in TG. JTT-130 did not induce hepatic lipid accumulation compared to controls. Cholesteryl ester transfer protein (CETP) activity was reduced in a dose dependent manner by increasing doses of MTPi and guinea pigs treated with atorvastatin had the lowest CETP activity (P < 0.01). In addition the number of molecules of cholesteryl ester in LDL and LDL diameter were lower in guinea pigs treated with atorvastatin. In contrast, hepatic enzymes involved in maintaining cholesterol homeostasis were not affected by drug treatment.

**Conclusion:**

These results suggest that JTT-130 could have potential clinical applications due to its plasma lipid lowering effects with no alterations in hepatic lipid concentrations.

## Background

Microsomal triglyceride transfer protein (MTP) is a resident protein in the lumen of endoplasmic reticulum and is primarily responsible for transfer of triglycerides (TG) and other lipids from their site of synthesis in the endoplasmic reticulum into the lumen during the assembly of very low density lipoprotein (VLDL) [1]. VLDL produced by the liver are the major source of LDL in plasma and elevated levels of LDL are associated with the development of atherosclerosis and cardiovascular disease (CVD). Increased total cholesterol and LDL cholesterol (LDL-C) are both considered primary risk factors for atherosclerosis [2,3]. To reduce CHD risk factors improvements in diet and exercise are primary recommendations however when plasma cholesterol concentrations reach a certain limit drug intervention is necessary. Statins, which are targeted to 3-hydroxy-3-methylglutaryl coenzyme A (HMG-CoA) reductase and are used extensively, are effective in lowering LDL-C, and somewhat effective in reducing plasma TG [4,5]. A number of studies done in the past have indicated that reduction in LDL-C values by using statins can significantly reduce the risk of CHD however a large population of patients still experience a clinical event [2,4,5]. Therefore, pharmaceutical companies are continuing to research other drug options to control hypercholesterolemia with the goal of developing a therapy for treating patients with dyslipidemias. Microsomal triglyceride transfer protein inhibitor (MTPi) is one such option. It is believed that blocking MTP will not only reduce plasma total and LDL cholesterol (LDL-C) but also plasma VLDL and TG by affecting the packaging and secretion of VLDL and chylomicrons. Certain animal and human studies [6,7] have shown that the inhibition of MTP blocks the hepatic secretion of VLDL and the intestinal secretion of chylomicrons. Consequently, this mechanism provides a highly efficacious pharmacological target for the lowering of LDL-C and reduction of postprandial lipemia. These effects could afford unprecedented benefit in the treatment of atherosclerosis and consequent cardiovascular disease. The promise of this therapeutic target has attracted widespread interest in the pharmaceutical industry.

This research study had a primary goal to evaluate whether (JTT-130), an MTPi reduces plasma cholesterol and triglyceride concentrations in male Hartley guinea pigs. Since JTT-130 is mainly targeted to the intestine, another main objective of this study was to evaluate whether this MPTi resulted in less hepatic lipid accumulation compared to other inhibitors [6,7]. Guinea pigs were used as the animal model for this study because of their similarities to humans in terms of hepatic cholesterol and lipoprotein metabolism. Previous studies done in our laboratory report that guinea pig serve as a good model for evaluating cholesterol lowering drugs [8-10].

## Methods

### Materials

Reagents were obtained from the following sources. JTT-130, the MTPi tested was provided by Akros Pharma Inc (Princeton, NJ). Enzymatic cholesterol and TG kits, cholesterol oxidase, cholesterol esterase and peroxidase were purchased from Roche-Diagnostics (Indianapolis, IN). Phospholipid and free cholesterol enzymatic kits were obtained from Wako Pure Chemical (Osaka, Japan). Quick-seal ultracentrifuge tubes were from Beckman (Palo Alto, CA). DL-hydroxy- [3-^14^C] methyl glutaryl coenzyme A (1.81 GBq/mmol), DL- [5-^3^H] mevalonic acid (370 GBq/mmol), cholesteryl- [1,2,6,7-^3^H] oleate (370 GBq/mmol), Aquasol, Liquiflor (toluene concentrate) and [^14^C] cholesterol were purchased from DuPont NEN (Boston, MA). Oleoyl- [1-^14^C] coenzyme A (1.8 GBq/mmol) and DL-3-hydroxy-3-methyl glutaryl coenzyme A were obtained from Amersham (Clearbrook, IL). Cholesteryl oleate, glucose-6-phosphate, glucose-6-phosphate dehydrogenase, nicotinamide adenine dinucleotide phosphate (NADP), sodium fluoride, Triton, bovine serum albumin and sucrose were obtained from Sigma Chemical (St. Louis, MO). Aluminum and glass silica gel plates were purchased from EM Science (Gibbstown, NJ).

### Diets

Diets were prepared and pelleted by Research Diets (New Brunswick, NJ). Isocaloric diets were designed to meet all the nutritional requirements for guinea pigs. The four diets had identical composition except for the type and dose of tested drug as indicated in Table 1. The amount of cholesterol in the diets was adjusted to be 0.08 g/100 g, an amount equivalent to 600 mg/day in the human diet [11].

**Table 1:** Composition of Control, low dose of the inhibitor (LDI), high dose of the inhibitor (HDI) and atorvastatin diets

Components	Control %	LDI %	HDI %	Atorvastatin %
Soybean protein	22.5	22.5	22.5	22.5
Methionine	0.5	0.5	0.5	0.5
Sucrose	25	25	25	25
Corn Starch	15	15	15	15
Fat mix^1^	15.1	15.1	15.1	15.1
Cellulose	10	10	10	10
Guar gum	2.5	2.5	2.5	2.5
Mineral Mix^2^	8.2	8.2	8.2	8.2
Vitamin Mix^2^	1.1	1.1	1.1	1.1
Cholesterol	0.08	0.08	0.08	0.08
JTT-130	0	0.0005	0.0015	0
Statin	0	0	0	0.05

^1 ^Fat mix for the diet contains olive oil-palm kernel oil-safflower oil (1:2:1.8), high in lauric and myristic acids.

^2 ^Mineral and vitamin mix adjusted to meet NRC requirements for guinea pigs. Detailed composition of the vitamin and mineral mix has been reported elsewhere (Fernandez et al. 1992b).

### Animals

Forty male guinea pigs (Harlan Sprague-Dawley, Hills), weighing 250–300 g, were randomly assigned to either a control, low dose of MTPi (LDI), high dose of MTPi (HDI) or an atorvastatin (AT) treatment (n = 10/group) for 4 weeks. Initially, all guinea pigs were fed the control diet for 3 weeks to raise plasma cholesterol concentrations. Two animals were housed per metal cage in a light cycle room (light from 0700–1900 h) and had free access to diets and water. Non-fasted guinea pigs were sacrificed by heart puncture after isoflurane anesthesia. Blood and livers were harvested for analysis and were stored at -80°C for further analysis. All animal experiments were conducted in accordance with U.S. Public Health Service/U.S. Department of Agriculture guidelines. Experimental protocols were approved by the University of Connecticut Institutional Care and Use Committee.

### Lipoprotein isolation

Plasma samples were collected from blood obtained by heart puncture from guinea pigs under anesthesia. A preservation cocktail of aprotonin, phenyl methyl sulfonyl fluoride and sodium azide was added to plasma samples to minimize changes in lipoprotein composition during isolation. Plasma was aliquoted for LCAT and CETP determinations, plasma lipid analysis and lipoprotein isolation.

Lipoproteins were isolated by sequential ultracentrifugation [12] in a LE-80K ultracentrifuge (Beckman Instruments, Palo Alto, CA). VLDL was isolated at *d *= 1.006 kg/L at 125,000 *g *at 15°C for 19 h in a Ti-50 rotor. LDL was isolated at *d *= 1.019-1.09 kg/L in quick-seal tubes at 15°C for 3 h at 200,000 *g *in a vertical Ti-65 rotor [13]. LDL samples were dialyzed in 0.9 g/L sodium chloride-0.1 g/L ethylene diamine tetra acetic acid (EDTA), pH 7.2, for 12 h and stored at 4°C for further analysis.

### Plasma and hepatic lipids

Plasma samples were analyzed for cholesterol and TG by enzymatic methods [14]. Hepatic total and free cholesterol and TG were determined according to the method by Carr et al. [15] following extraction of hepatic lipids with chloroform-methanol 2:1. Cholesteryl ester concentrations were calculated by subtracting free from total cholesterol.

### Lipoprotein characterization

VLDL and LDL composition was calculated by determining free and esterified cholesterol [14], protein by a modified Lowry method [16], and TG and phospholipids by enzymatic kits. VLDL apo B was selectively precipitated with isopropanol [17]. The number of constituent molecules of LDL was calculated on the basis of one apo B per particle with a molecular mass of 412000 kD[18]. The molecular weights were 885.4, 386.6, 645 and 734 for TG, free and esterified cholesterol, and phospholipids, respectively [19]. LDL diameters were calculated according to Van Heek et al [20]. HDL cholesterol was also determined according to Warnick et al, with a modification, which consisted of using 2 mol/L MgCl_2 _for precipitation of apo-B containing lipoproteins [13].

### Lecithin Cholesterol Acyltransferase (LCAT) and Cholesterol Ester Transfer Protein (CETP) determinations in plasma

LCAT and CETP activities were determined according to Ogawa & Fielding [21]. Physiological CETP activity was determined without inhibiting LCAT activity by measuring the mass transfer of cholesterol ester between HDL and apo B containing lipoproteins. Samples were incubated at 37°C for 6 h in a shaking water bath and total and free plasma cholesterol and HDL cholesterol were measured. LCAT activity was determined by mass analysis of the decrease in plasma free cholesterol between 0 and 6 h at 37°C. Assays were carried out concurrently with measurements of CETP. Both of these methods have been well-standardized for guinea pig plasma [22].

### Hepatic microsome isolation

Hepatic microsomes were isolated as described previously [8]. Briefly a microsomal fraction was isolated by two 25-min centrifugations at 10,000 *g *(JA-20 rotor, J2-21) followed by ultracentrifugation at 100,000 *g *in a Ti-50 rotor at 4°C for 1 hour. Microsomes were resuspended in the homogenization buffer and centrifuged for an additional hour at 100,000 *g*. After centrifugation, microsomal pellets were homogenized and stored at -70°C.

### Hepatic HMG-CoA reductase assay

The activity of microsomal HMG-CoA reductase (E.C. 1.1.1.34) was measured in hepatic microsomes as described by Shapiro et al. [23]. HMG-CoA reductase activity was expressed as pmol of [^14^C] mevalonate produced per min per mg microsomal protein. Recoveries of [^3^H] mevalonate ranged from 60–90%.

### Hepatic Acyl CoA Cholesteryl Acyltransferase (ACAT) activity

Hepatic ACAT (E.C. 2.3.1.26) activity was measured by the incorporation of [^14^C] oleoyl CoA in cholesteryl ester in hepatic microsomes by preincubating 0.8–1 mg of microsomal protein per assay with 84 g/L albumin and buffer for microsomal isolation [24]. Recoveries of [^3^H] cholesteryl oleate were between 70–90%.

### Hepatic cholesterol 7α-hydroxylase activity

Cholesterol 7α-hydroxylase (E.C. 1.14.13.7) activity was measured according to the method modified by Jelinik et al [25]. [^14^C] cholesterol was used as a substrate and delivered as cholesterol-phosphatidylcholine liposomes (1:8 by weight) prepared by sonication. An NADPH-regenerating system (glucose-6-phosphate dehydrogenase, NADP, and glucose-6-phosphate) was included in the assay as a source of NADPH.

### Statistical analysis

One-way analysis of variance (ANOVA) (SSPS for Windows version 12) was used to evaluate significant differences among groups in regards to plasma and hepatic lipids, LDL composition, hepatic enzyme activities and LCAT and CETP activities. The LSD post hoc test was used to evaluate the differences among groups. Data are presented as the mean ± SD. Differences were considered significant at P < 0.05.

## Results

### Plasma lipids and lipoproteins

**Figure 1:**
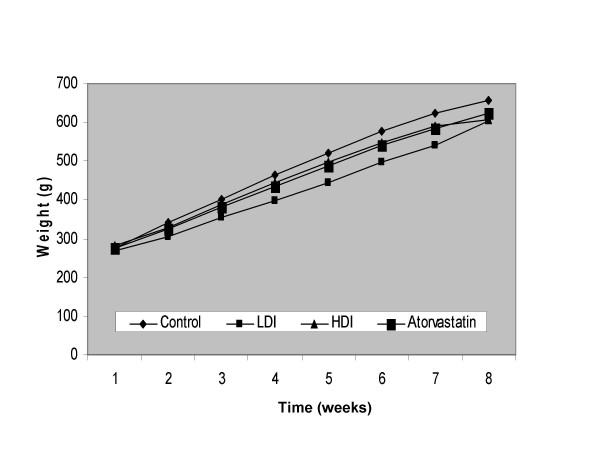
Weight gain of guinea pigs treated with control, low dose, high dose of JTT-130 and atorvastatin.

No difference in weight gain overtime was observed in guinea pigs fed the different test diets (Fig 1), indicating that animals consumed comparable amounts of their respective test diets. After feeding the test diets for a period of four weeks, blood was isolated and plasma was analyzed for cholesterol and TG concentrations. The two doses of MTPi evaluated, low dose (LDI) and high dose (HDI) decreased plasma total cholesterol values significantly by 19.2% (P < 0.01) as compared to the control animals (Table 2). There was no significant difference between the two doses of MTPi tested. Atorvastatin, which was used as a positive control, led to a significantly robust decrease in plasma total cholesterol values of 46%, which was significantly different (P < 0.01) from the two MTPi doses used. Plasma TG values were also significantly lower in LDI (50.8%) and HDI (45.3%) when compared to their control counterparts whereas atorvastatin (AT) treatment resulted in the maximum decrease of plasma TG (Table 2).

**Table 2:** Plasma total cholesterol (TC), triglycerides (TG), VLDL-C, LDL-C and HDL-C of guinea pigs fed a control diet, low dose MTPi (LDI), high dose MTPi (HDI) or atorvastatin

Diets	TC	TG	VLDL-C	LDL-C	HDL-C
			(mg/dL)		
Control (10)	146.9 ± 42.2^a^	135.8 ± 118.9^a^	8.1 ± 5.5^ab^	123.9 ± 43.5^a^	13.6 ± 4.5^a^
LDI (10)	116.9 ± 29.7^b^	66.7 ± 29.6^b^	3.7 ± 3.0^a^	93.3 ± 26.2^b^	18.5 ± 8.0^a^
HDI (9)	116.1 ± 23.3^b^	74.3 ± 31.7^b^	9.0 ± 8.6^b^	90.5 ± 28.8^b^	15.6 ± 7.1^a^
Atorvastatin (9)	76.5 ± 29.8^c^	49.4 ± 32.2^c^	2.9 ± 2.6^a^	59.4 ± 29.3^c^	14.1 ± 5.6^a^

^1^Data are presented as mean ± SD for the number of guinea pigs indicated in parenthesis. Numbers in a column with different superscripts are considered significantly different (P < 0.01) as determined by one way ANOVA and LSD as a post-hoc test

The changes in plasma TC values were mostly due to decreases in the cholesterol carried by LDL. LDL-C was also significantly decreased (24.7% & 26.9%) by the MTPi diets tested. There were no major differences between these two doses for plasma lipid parameters except for VLDL-C, which were significantly higher when compared to the low dose of the drug. No significant differences were observed for HDL-C values with MTPi or with AT (Table 2).

### LDL size and composition

No significant effect of MTPi on the number of CE molecules or on the size of LDL particle was found with any of the two doses tested as compared to their control counterparts (Table 3). AT treatment reduced the number of esterified cholesterol molecules (45%) as well as decreased the size of LDL particle (30%) (Table 3).

**Table 3:** Number of molecules of cholesteryl ester (CE), LDL diameter and LCAT and CETP activities of guinea pigs fed a control diet, low dose MTPi (LDI), high dose MTPi (HDI) or atorvastatin.

Diets	CE molecules	LDL diameter	LCAT	CETP
		nanometers	(pmol/min.mg)	
Control (n = 10)	1993 ± 422^a^	16.47 ± 3.63^a^	19.6 ± 11.0^a^	36.1 ± 12.5^a^
LDI (n= 10)	2072 ± 536^a^	16.67 ± 3.05^a^	14.4 ± 6.4^a^	31.0 ± 21.1^a^
HDI (n = 9)	2026 ± 132.5^a^	16.58 ± 7.85^a^	14.6 ± 8.5^a^	19.4 ± 13.0^ab^
Atorvastatin (n = 9)	1080 ± 1092^b^	11.52 ± 2.69^b^	13.4 ± 10.9^a^	12.8 ± 4.2^b^

^1^Data are presented as mean ± SD for the number of guinea pigs indicated in parenthesis. Numbers in a column with different superscripts are considered significantly different (P < 0.01) as determined by one way ANOVA and LSD as post hoc test.

### LCAT and CETP activities

Table 3 also summarizes the activities of these two proteins, which play a major role in the intravascular processing of plasma cholesterol. There were no significant differences in LCAT activity when comparing MTPi or statin groups to the control group. However, HDI decreased the activity of CETP, which was comparable to the atorvastatin treated group (P < 0.01).

### Hepatic lipids and enzymes

No significant changes were found in hepatic total cholesterol, free cholesterol, cholesteyl ester or TG values in any of the four treatments (Table 4). Results suggest that MTPi did not lead to lipid accumulation in the liver, as there was no significant difference between the two doses of MTPi tested with control or with atorvastatin group. Similarly no significant changes were observed in any of the three regulatory hepatic enzymes involved, namely CYP7, ACAT and HMG-CoA Reductase (Table 5).

**Table 4:** Hepatic total cholesterol (TC), free cholesterol (FC) cholesteryl ester (CE) and TG of guinea pigs fed a control diet, low dose MTPi (LDI), high dose MTPi (HDI) or atorvastatin^1^.

Diets	TC	FC	CE	TG
		(mg/g)		
Control (n = 10)	1.20 ± 0.53	1.00 ± 0.44	0.20 ± 0.23	16.2 ± 2.8
LDI (n = 10)	1.47 ± 0.40	1.24 ± 0.35	0.23 ± 0.11	13.9 ± 9.4
HDI (n = 9)	1.42 ± 0.66	0.98 ± 0.39	0.44 ± 0.58	13.5 ± 7.8
Atorvastatin (n = 9)	1.71 ± 0.76	1.31 ± 0.56	0.40 ± 0.39	18.8 ± 10.8

^1^Data are presented as mean ± SD for n = the number of guinea pigs indicated in parenthesis.

**Table 5:** Hepatic HMG-CoA reductase, ACAT and cholesterol 7α-hydroxylase activities (CYP7) guinea pigs fed a control diet, low dose MTPi (LDI), high dose MTPi (HDI) or atorvastatin^1^

Diets	HMG-CoA Reductase	ACAT	CYP7
		(pmol/min.mg)	
Control	1.8 ± 0.8	0.8 ± 0.4	1.7 ± 1.8
LDI	2.7 ± 0.8	2.2 ± 0.9	1.2 ± 0.5
HDI	2.5 ± 1.4	3.5 ± 2.4	0.6 ± 0.7
Atorvastatin	3.4 ± 2.9	4.4 ± 3.1	0.7 ± 0.4

^1^Data are presented as mean ± SD for n = 3–7 guinea pigs per group.

## Discussion

In this study we were able to demonstrate that JTT-130, the MTPi tested has the potential to decrease the prime risk factors of cardiovascular disease, namely plasma TG and LDL-C concentrations in guinea pigs. The novelty of the drug tested is that there was no significant accumulation of lipid in the liver as seen in some other studies done with other MTPi [26,27].

### Drug treatment and hepatic lipids

Previous studies evaluating MTPi have shown increase in the lipid content of the liver [7,26,27]. Chandler et al [7] treated Hep-G2 cells with CP-346086, another MTPi, for a period of two weeks. They reported that in addition to decreasing plasma TC, LDL-C, VLDL-C and TG values, this treatment also increased hepatic and intestinal TG when the MTPi was administered with food and when it was dosed away from meals, only hepatic TG were influenced. In contrast, the major finding of this study is that the MTPi tested did not lead to any fat accumulation in the liver as confirmed by no significant changes found in the hepatic lipid content as compared to their control counterparts. The main reason for these differences between MTPi could be that the main target of JTT-130 was the intestine. Because of this, we speculate that due to MTP inhibition, less TG were transferred to the chylomicron particle being packaged in the intestine. As a result a lower concentration of TG was taken up by the hepatocytes through the chylomicron remnant. Thus the VLDL particles secreted from the liver had lower concentrations of TG molecules due to the major inhibitory effect of the MTPi in the intestine. Because there were no significant changes in hepatic cholesterol concentrations, we did not find any significant differences in hepatic enzyme activities. Similar to the study by Conde et al. [9] in atorvastatin treated guinea pigs with 0.015% atorvastatin, there were no significant differences in hepatic cholesterol concentrations when compared with a control group. However, significant differences in hepatic esterified cholesterol were observed when guinea pigs were treated with a higher dose of the statin (0.05%) [9].

### Drug treatment and plasma lipids and lipoproteins

Abetalipoproteinemia, a genetic disorder characterized by low plasma cholesterol and TG levels, is caused by a functional deficiency of MTP. Absence of lipid transfer activity in the microsomes of abetalipoproteinemia patients established its pivotal function in lipoprotein assembly[1]. This finding led to the suggestion that MTP inhibition could be used as a possible lipid lowering therapy. Further evidence was obtained from a cell culture study in which researchers [28] proved that MTP is limiting in the production of apo B containing lipoproteins. Another study [6] further confirmed this finding using heterozygous MTP knockout mice which had 20% less plasma total cholesterol levels compared to wild type mice fed high fat diets; however, they did not find any significant differences in plasma TG concentrations. In our study, we have demonstrated that animals treated with MTPi had not only lower plasma TC and LDL-C but also significant reductions in plasma TG. It is possible that the VLDL particle secreted by the liver was more readily catabolized and therefore there was less conversion to LDL, which contributed to the hypocholesterolemic effects of the MTPi. Conde et al. [9] demonstrated that there was a significant reduction in plasma TG in guinea pigs treated with atorvastatin when compared to controls. This was partly explained by lower secretion of VLDL particles and by increases in the LDL receptor [9], which could have contributed to the faster removal of VLDL particles. A similar mechanism may have taken place with the MTPi. By blocking MTP, JTT-130 reduced the secretion of VLDL particles, and therefore, the formation of LDL in the plasma.

There was a dose response in CETP activity with JTT-130, and in addition, guinea pigs treated with atorvastatin exhibited decreased activity of this transfer protein. The main function of CETP is to contribute to the reverse cholesterol pathway by transferring cholesteryl ester from HDL to the apo B containing lipoproteins [29]. However, this action prolongs the residence time of CE in LDL and increases the possibility of its deposition in the arterial wall. Thus lower CETP activity has been associated with decreased atherogenesis in animal studies [30]. Therefore the lowering of CETP activity by drug treatment can be considered beneficial.

Results from this study indicate that JTT-130 has the potential to reduce the primary risk factors for coronary heart disease. While these results are quite promising, more studies are needed to clarify the possibility of adverse effects including steatorhea, fat malabsorption and fat-soluble vitamin absorption. Although the lipid lowering effects were not as pronounced as those observed with atorvastain, the doses of MTPi used in the current study were lower than the doses of atorvastatin. It is possible that using JTT-130 in combination with statins could reduce the wide array of adverse effects associated with reductase inhibitors [31]. This study also demonstrates that MTP inhibitor which is mainly targeted towards the intestine may open a new avenue for treatment of hyperlipidemic patients who are at high risk for cardiovascular diseases.

## List of abbreviations used

ACAT: acyl CoA cholesteryl acyl transferase; AT: atorvastatin; CETP: cholesterol ester transfer protein; CHD: coronary heart disease; CYP7: cholesterol 7α-hydroxylase; HDi: high dose of the inhibitor; HDL-C: HDL cholesterol; HMG-CoA; 3-hydroxy-3methyl glutaryl Conezyme A; LCAT: lecithin cholesterol acyltransferase; LDi: low dose of the inhibitor; LDL-C: LDL cholesterol; MTP: microsomal transfer protein; MTPi: microsomal transfer protein inhibitor; NADP: nicotinamide adenenine dinucleotide phosphate; TG: triglycerides; VLDL: very low density lipoprotein.

## Competing interests

Authors received funding from Akros Pharma Inc. (Princeton, NJ) to carry out the studies presented in this manuscript.

## Authors' contributions

DA did the assays, wrote the manuscript and participated in the interpretation of data; KLW: assisted in the assays for plasma lipids, CETP and LCAT; TLZ: assisted in the determination of ACAT activity and participated in data interpretation, SS: assisted in taking care of guinea pigs, isolation of microsomes and data interpretation; MVJ assisted in the determination of CYP7 and in data interpretation and MLF designed the experiment, evaluated the results, interpreted the data and participated in manuscript preparation.

## Pre-publication history

The pre-publication history for this paper can be accessed here:


